# The validity, reliability and feasibility of four instruments for assessing the consciousness of stroke patients in a neurological intensive care unit compared

**DOI:** 10.1186/s12874-022-01580-2

**Published:** 2022-04-08

**Authors:** Xiaoxiang Yan, Lingjun Xiao, Meixin Liao, Jiajian Huang, Zhijie He, Tiebin Yan

**Affiliations:** 1 Intensive Care Unit, Department of Neurology, First Affiliated Hospital, Anhui Medical University, Hefei, China; 2 Intensive Care Unit, Department of Critical Care Medicine, Sun Yat-sen Memorial Hospital, Sun Yat-sen University, Guangzhou, China; 3 Department of Rehabilitation Medicine, Sun Yat-sen Memorial Hospital, Sun Yat-sen University, Guangzhou, China

**Keywords:** Consciousness, Intensive care, Assessment scales, Richmond Agitation-sedation Scale, Motor activity assessment scale, Glasgow Coma Scale, Sedation-agitation scale

## Abstract

**Background:**

Early rehabilitation is the foundation for recovery for those admitted to an intensive care unit. Appropriate assessment of consciousness is needed before any rehabilitative intervention begins.

**Methods:**

This prospective study compared the validity, reliability and applicability of the sedation-agitation scale, the Richmond Agitation-Sedation Scale, the motor activity assessment scale and the Glasgow Coma Scale in a working neurological intensive care unit. Eighty-three stroke patients were assessed with the four scales by the same 3 raters acting independently: a senior physician, a senior therapist and a trainee. That generated 996 assessment records for comparison.

**Results:**

Good agreement (*r*=0.98–0.99) was found among the sedation-agitation scale, the Richmond Agitation-Sedation Scale, the motor activity assessment scale scores, but the Glasgow Coma Scale ratings correlated less well (*r*=0.72–0.76) with the others. Consistent results were also found among the three raters. After stratification of the ratings by age, gender, level of consciousness and Acute Physiology and Chronic Health Evaluation score, the scales reported significant differences among the levels of consciousness and among those with different Acute Physiology and Chronic Health Evaluation results, but not with different age or gender strata.

**Conclusions:**

The four instruments tested are all reliable enough and feasible for use as a tool for consciousness screening in a neurological intensive care unit.

## Background

Human consciousness originates in the brain, and if damage to the brain impairs consciousness, behavior is usually affected. Disordered consciousness is common after traumatic brain injury or a stroke. Stroke appears to be the most common cause of non-traumatic coma in the emergency department [1]. With the increasing incidence of stroke worldwide [2], more stroke victims are being managed initially in neurological intensive care units (NICUs) where they can receive more specific and more professional management with better outcomes [3]. Those with disordered consciousness often demonstrate somewhat impaired ability to receive, process, store and recall information [4–6]. Clearly, disturbed consciousness can delay the recovery of a damaged brain and impact a person’s active participation even after the underlying disease is medically stable [7].

Much has been published about evaluating consciousness following traumatic brain injury [8], but little about evaluating consciousness following a stroke [9]. This despite that fact that stroke is one of the most common causes of disability [2]. Evidence has strongly supported early rehabilitation after a stroke and demonstrated that early intervention tends to lead to better outcomes [10, 11].

Rehabilitation is certainly recommended for patients with disordered consciousness [12, 13]. Applying techniques such as bed positioning, pulmonary exercise and physical modalities early in the NICU is now widely accepted [14, 15]. But before any rehabilitative intervention begins, the rehabilitation team must ascertain the patient’s level of consciousness. That is essential for making appropriate decisions about what kind of intervention, either active or passive, and also how much intervention would be appropriate. It is therefore important to evaluate basic information about consciousness before deciding when to start rehabilitation and at what intensity [13, 16]. The goals and content of a patient’s rehabilitation program should be set with reference to the results of a consciousness level assessment [17]. Initial impairment of consciousness has been shown to modify the effects of early, goal-directed mobilization therapy, though even then such mobilization is usually effective and not harmful [7]. A group led by Green has suggested that when mobilizing subjects in an ICU, the first step should be to assess the subject’s level of alertness and ability to follow the instructions. They used the Richmond Agitation-Sedation Scale in their study and found that if the subject was unable to follow commands (a Richmond Agitation-Sedation Scale score of < –1), only passive mobilization was suitable. Otherwise, active mobilization should be considered [18]. Therefore, an appropriate rehabilitation program should be based on the patient’s level of consciousness and modified depending on the level to maximize the effectiveness of treatment and help to minimize any negative effects.

There are some specific consciousness measuring tools and scales. They are mainly used in the ICU [19]. From a clinical point of view however, they need to be further tested for validity, reliability and feasibility if they are to be applied routinely with stroke survivors in the NICU. Ramsay and his colleagues introduced a 6-point scale forty years ago [20]. Since then, other assessment scales such as Sessler’s Richmond Agitation-Sedation scale (RASS) [21], the sedation agitation scale (SAS) developed by Riker’s group [22] and the motor activity assessment scale (MAAS) from a group led by Devlin [23] have been developed to evaluate consciousness in an ICU. Groups led by Ryder [24] and by Zhong [25] have since drawn similar conclusions about the reliability of the SAS. Since the RASS, MASS and SAS are often used in an ICU [26], they should, theoretically, also be helpful when used in an NICU before starting a rehabilitation program.

This study was designed to evaluate for stroke rehabilitation the scales that are commonly applied in traumatic brain injury cases. The study focused on a Chinese population, where early rehabilitation has developed quickly in recent years and rehabilitation team members are often involved in bedside treatment in the NICU. Scales are, however, are rarely used to judge the consciousness of NICU patients in China. One of the main reasons is that clinicians are unsure about their applicability with stroke survivors. Few if any treatments explicitly evaluate the patient’s consciousness level and adjust the program accordingly. The Glasgow Coma Scale (GCS) is the scale most commonly applied in NICUs in China, so one goal of this study was to compare that scale’s validity with those of the RASS, MAAS and SAS, taking the GCS as the gold standard for stroke treatment in an NICU. The study also examined inter-rater agreement. That was done using a team of three—a senior physician, a senior physiotherapist and a physiotherapy trainee. The scales’ clinical feasibility was also evaluated, seeking the most appropriate tool for evaluating subjects in China’s NICUs. This has been the first study to do so with a Chinese population.

## Methods

### Study design and setting

This was a prospective study in which the validity, reliability and feasibility of the four scales for use in a Chinese NICU were scored by three raters. The senior physician had worked in NICUs for seven years; the senior physiotherapist had worked for more than ten years with subjects in NICUs; the third rater was a final year rehabilitation student with little NICU experience. The study protocol was approved by the ethics committee of the Sun Yat-sen Memorial Hospital of Sun Yat-sen University in Guangzhou, China where the whole study was carried out between July 1^st^ and December 30^th^ of 2019. This study was not registered because behavior was only observed and evaluated as is routinely done in any NICU without any intervention or follow-up assessment.

### Subjects

This study was carried out in an NICU where the majority of the patients were stroke survivors. It treated few if any traumatic brain injuries, so the inclusion criteria specified subjects who had suffered a stroke diagnosed by brain CT or MRI as infarction or hemorrhage, aged 18 years or older. Patients were excluded if they had suffered a traumatic brain injury, had any neuromuscular blockage or quadriplegia, were subject to contact or airborne isolation precautions, had impaired hearing or vision, or did not speak Chinese. Consciousness was classified using the levels proposed by Peng and his colleagues [27].

### Outcome measures

The RASS, MAAS, SAS and GCS were all administered to each subject to generate head-to-head comparisons. None is normally considered to be consistently superior [28]. The RASS is a subjective scale which makes it convenient in clinical practice. It can be administered within one minute based on observation, response to verbal stimulation and response to physical stimulation. It has a 10-point numerical scale graded from -5 to +4 with four levels of agitation (+1 to +4), one level for calm and alert (0), and 5 levels of sedation (–1 to –5) [21, 29]. Using the RASS is not time-consuming and it is used in various settings from pediatrics to the emergency room [29, 30], consistently showing good validity. The Motor Activity Assessment Scale and the SAS use a 7-point scale ranging from 0 to 6. The MAAS mostly focuses on a subject’s responses to noxious stimuli and defines two levels: response or no reaction [23]. Samuelson’s group found that MAAS scores can usually predict memory recall in an NICU [31]. The GCS developed by Teasdale is the one most widely used in China for evaluating consciousness. It scores eye opening, verbal and motor reactions [32].

Before the main study, the three raters were trained on the four scales in three sessions so that they fully understood the content and how to use them. The first 1-hour session was devoted to learning and understanding the contents of the four scales. The three raters had some discussion about them during and after the training. The second session was 2 hours devoted to practicing with the scales, again with discussion. In the third training session a pilot study was conducted using 5 actual stroke patients in an NICU.

During the main study, each subject’s age, gender, etiology at admission, acute physiology and chronic health evaluation (APACHE II) score were recorded, as well as whether or not they were intubated, had received ventilation or had been sedated. The APACHE II evaluation was first published in 1985 and designed as a severity of disease classification system for use in ICUs. It has demonstrated good validity [33]. It is sometimes applied with stroke patients in ICUs [34]. APACHE II was therefore one of the study’s evaluation tools. A few published studies of stroke survivors have found that APACHE II scores are closed related to the severity of the disease, with higher the APACHE II scores indicating more serious disease [33, 34].

**Fig. 1:**
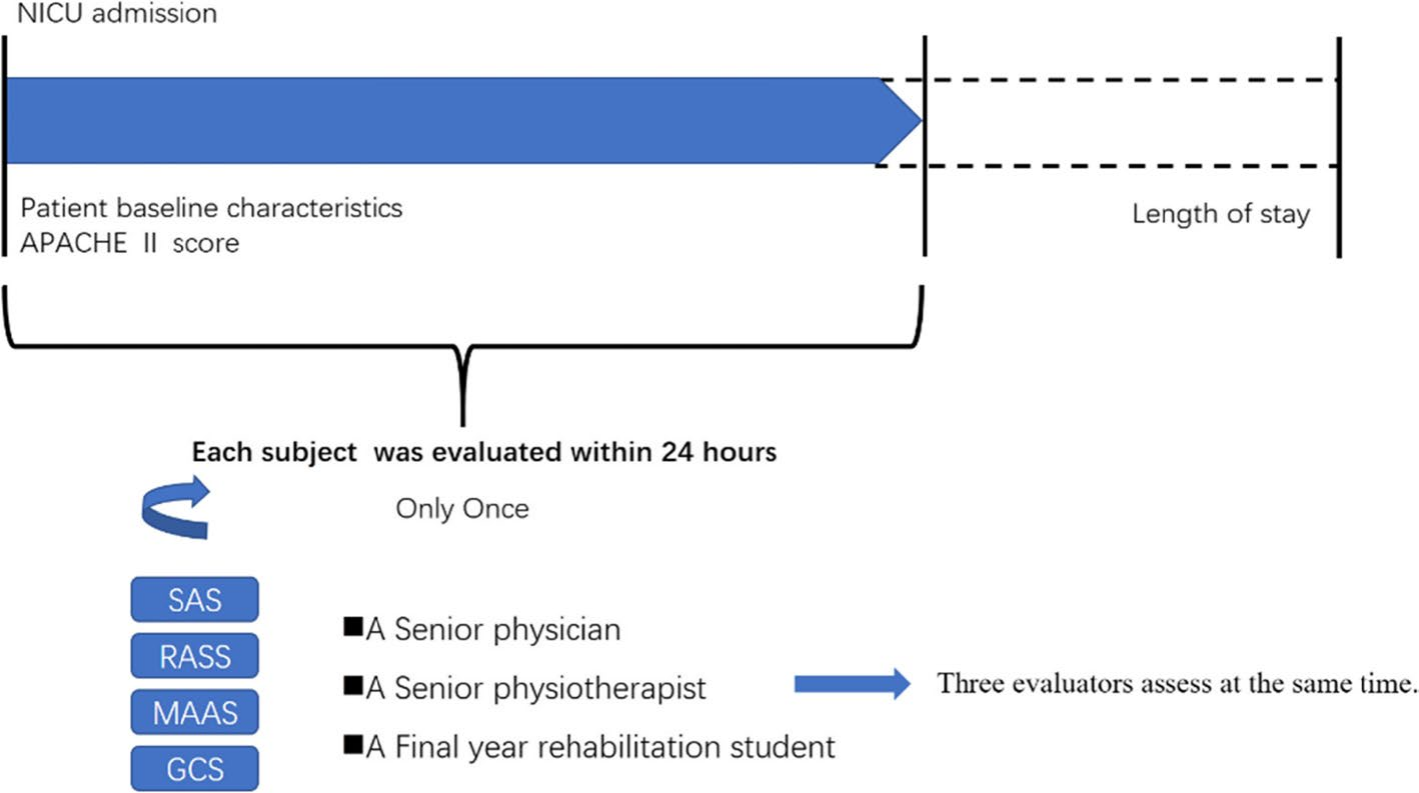
The assessment and data collection schedule of the study. APACHE: Acute Physiology and Chronic Health Evaluation, SAS: Sedation-Agitation Scale, RASS: Richmond Agitation-Sedation Scale, MAAS: Motor Activity Assessment Scale, GCS: Glasgow Coma Scale

Each subject was evaluated only once within 24 hours of being admitted to the NICU. During the evaluation the senior physician communicated with and stimulated each subject first while the other two evaluators looked on. The scale ratings were then scored and recorded on the scales’ respective assessment sheets by the three raters separately and concurrently. There was no communication among the raters during or after the scoring procedure. The data were entered into the computer by others, so no investigator knew the others’ scoring of each subject. Figure 1 shows the assessment and data collection schedule.

### Statistical analysis

The statistical analyses were performed after all the information had been collected and scoring was complete. The data were analyzed using version 21.0 of the Statistical Package for the Social Sciences (SPSS). Descriptive statistics were compiled summarizing the subjects’ demographic and clinical characteristics. The data were expressed as mean ± standard deviation (SD), medians and inter-quartile ranges.

In order to minimize the deviation of the four scales caused by baseline characteristics, the significant difference with age and the APACHE II scores were compared with stratification following published studies [33–35] and using their medians. Age was stratified as younger than 60 vs 60 or older [35]. The APACHE II scores were divided into those 10 or less and those >10, and other results were compared between the low-scoring and high-scoring groups.

In addition, the non-parametric Wilcoxon signed-rank test was applied to assess the significance of any differences in scores between the different strata. Weighted kappa (κ_w_) values and their 95% confidence intervals were used to evaluate inter-rater agreement. Kappa values of more than 0.8 were considered to indicate “very good” agreement while more than 0.6 was considered “substantial” agreement [36]. Spearman rank correlation coefficients were computed, but no multivariate regression analyses were performed. A value of *p* ≤ 0.05 was considered as indicating statistical significance.

## Results

**Fig. 2:**
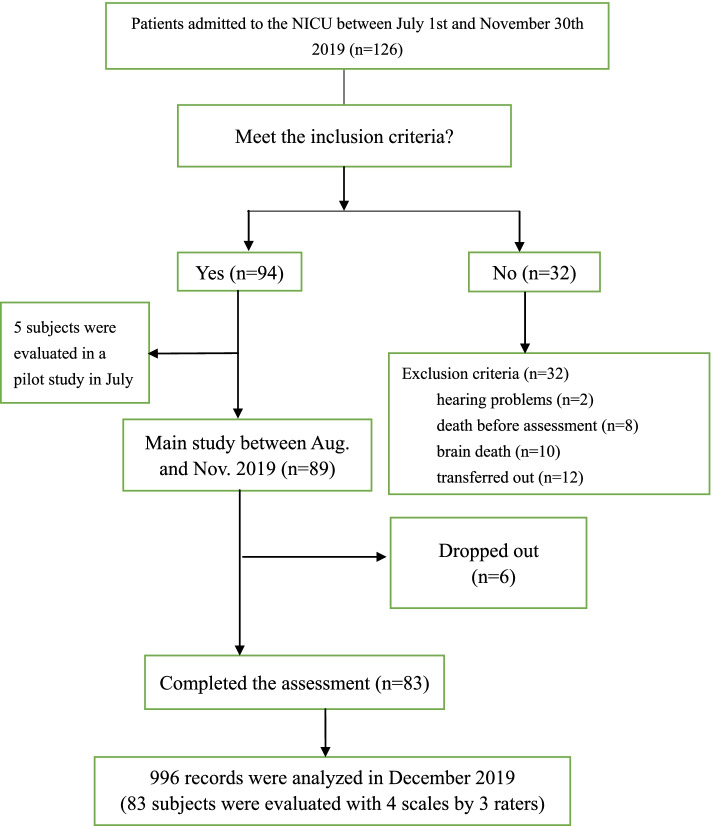
Study protocol for evaluation with the 4 scales by 3 assessors

One hundred and twenty-six persons were admitted to the NICU between August 1^st^ and November 30^th^. Ninety-four of them were deemed eligible for the study. Apart from the 5 cases in the pilot study in July, 6 were not assessed because they were admitted during a national holiday when the assessors were not on duty. Finally, a total of 996 records were generated: 3 raters describing 83 cases using the 4 scales. Another thirty-two were excluded due to hearing problems (*n*=2), deep coma (*n*=10) or death (*n*=8), and twelve were transferred to other departments before they could be assessed. Figure 2 shows the study’s protocol.

The ages of the 83 subjects assessed ranged from 19 to 93 years (mean value 57.3). Twenty-seven (32.53%) were female. The reasons for admission were either ischemic stroke (*n*=65), or after surgery following a hemorrhage (*n*=18). Seventy-eight of the subjects (93.98%) had been intubated and seventy-seven had received mechanical ventilation. In terms of Peng’s four consciousness levels [27], fifty-one subjects were classified as alert at the time of assessment, 15 as drowsy, 4 as stuporous and the other 13 were adjudged to be comatose based on behavior. The subjects’ baseline characteristics are summarized in Table 1.

**Table 1:** Baseline characteristics of the subjects studied

Characteristic	
Age (mean ± SD), years	57.31±17.91
Female, n (%)	27(32.53)
Type of Stroke, n (%)	
Ischemia	65(78.31)
Hemorrhage	18(21.69)
Mechanical ventilation, n (%)	77(92.77)
Intubation, n (%)	78(93.98)
Sedated n(%)^a^	40(48.19)
Consciousness , n (%)	
Alert	51(61.45)
Drowsy	15(18.07)
Stuporous	4(1.82)
Comatose	13(15.66)
APACHE II score (mean ± SD)	12.08±6.75

^a^indicates that the patient had taken sedative medication when assessed by the raters

Table 2 shows the data stratified by age, gender, and APACHE II score. There were no significant differences between the age and gender strata, indicating that neither age nor gender had a meaningful impact on the ratings. The APACHE II score strata did, however, show some significant differences in the scores with the four instruments.

**Table 2:** The Distribution of the ratings with different types of subjects

	**Number**	**RASS** Me [LQ; UQ]	**SAS** Me [LQ; UQ]	**GCS** Me [LQ; UQ]	**MAAS** Me [LQ; UQ]
All	83	0[–2;0]	4[3;4]	11[10;15]	3[2;3]
Age, yr					
<60	40	0[–2;0]	4[3;4]	11[10;15]	3[2;3]
** ≥**60	43	0[–3;0]	4[2;4]	11[7;15]	3[2;3]
* p*		0.40	0.40	0.45	0.34
Gender					
Male	56	0[–2;0]	4[3;4]	11[10;15]	3[2;3]
Female	27	0[–2;0]	4[3;4]	11[10;15]	3[2;3]
* p*		0.30	0.82	0.35	0.30
APACHE II Score					
≤10	45	0[0;0]	4[4;4]	10[11;15]	3[3;3]
>10	38	–2[–4;0]	3[2;4]	10[6;11]	2[1;3]
* p*		≤0.001	≤0.001	≤0.001	≤0.001

*Abbreviations*: *Me* Median, *LQ* Lower Quartile, *UQ* Upper Quartile

The scores using each scale are presented as Me [LQ; UQ] for each group. The *p* values test the significance of the Me differences using the non-parametric Wilcoxon signed-rank test between the groups

Spearman rank correlation coefficients were computed to quantify the agreement among the scales and among the three raters, as well as agreement for all of the ratings together. Those results are shown in Table 3. An interesting finding is the strong correlations and significant difference between the RASS and SAS scores (*r*=0.99, *p*≤0.001), the RASS and MASS scores (*r*=0.99, *p*≤0.001), and the SAS and MASS scores (*r*=0.99, *p*≤0.001). Even more interesting is the weaker correlations between the GCS scores and those using the other three scales. The correlation coefficients of all three raters were very similar.

**Table 3:** Spearman correlation coefficients between the scales’ ratings^a^

	Physician r(95%CI)	Physiotherapist r(95% CI)	Trainee r(95% CI)	Overall r(95% CI)
RASS versus SAS	0.99(0.99–1.00)	0.99 (0.99–1.00)	0.99 (0.99–1.00)	0.99(0.99–1.00)
RASS versus GCS	0.74(0.62–0.84)	0.75(0.61–0.84)	0.74 (0.61–0.83)	0.74(0.67–0.80)
RASS versus MAAS	0.99(0.98–0.99)	0.99 (0.99–1.00)	0.98 (0.97–0.99)	0.99 (0.98–0.99)
SAS versus GCS	0.74(0.62–0.84)	0.74(0.61–0.83)	0.74 (0.61–0.83)	0.74 (0.67–0.80)
SAS versus MAAS	0.99(0.98–0.99)	0.99 (0.99–1.00)	0.98 (0.97–0.99)	0.99(0.98–0.99)
GCS versus MAAS	0.76(0.63–0.85)	0.73 (0.60–0.83)	0.72(0.57–0.82)	0.74(0.66–0.79)

CI denotes confidence intervals. r is Spearman’s ranked correlation coefficient. RASS, SAS, GCS, MAAS respectively represent the Richmond Agitation-Sedation Scale, the sedation-agitation scale, the Glasgow Coma Scale and the motor activity assessment scale

^a^indicates all *p*≤0.001

Table 4 shows that the different assessors and scales produced similar overall scores and 95% confidence intervals. The weighted kappas for the RASS, SAS and GCS scores were all over 0.96, and their 95% CIs were between 0.92 and 1.0, though that did not extend to the MASS scores. The results of the MAAS scores had κ_w_ values less than 0.90 and their 95% CIs ranged from 0.75 to 1.0 when compared with the results using the other three scales.

**Table 4:** Inter-rater reliability of the four sedation-agitation scales

	RASS κ_w_ (95% CI)	SAS κ_w_ (95% CI)	GCS κ_w_ (95% CI)	MAAS κ_w_ (95% CI)
physician vs physiotherapist	0.99(0.98–1.00)	0.98(0.95–1.00)	0.98(0.96–0.99)	0.92(0.80–1.00)
physician vs trainee	0.96(0.93–0.99)	0.98(0.95–1.00)	0.97(0.95–0.99)	0.88(0.75–1.00)
physiotherapist vs trainee	0.96(0.93–0.99)	0.96(0.92–0.99)	0.98(0.97–1.00)	0.95(0.91–0.99)

The numbers in the table are stratified results except for overall

## Discussion

This study applied a team model with three raters testing four scales commonly used with traumatic brain injury in the clinic for their applicability to stroke patients. It found excellent agreement among the four scales and among the ratings of the three rather different assessors, indicating that these four scales are reliable and feasible when applied with stroke survivors, at least among a Chinese population. Of course, following each scale’s application guidelines is necessary to guarantee reliable results [37], nevertheless, this study has demonstrated that even a final year student of physiotherapy was able to assess effectively with simple training.

Today, evidence from randomized clinical trials has strongly supported early rehabilitation in the NICU [38–41]. With the increased incidence of stroke internationally [2], facilitating recovery following a stroke is attracting more attention from clinicians. How to speed up the recovery process is a key topic. Early rehabilitation is known to increase chances of a good recovery with a shorter hospital stay and less residual impairment [12] .The first step in such rehabilitation should be to assess the patient’s level of consciousness to guide whether passive or active rehabilitation techniques would be more appropriate [42, 43]. The suitability and reliability of the scale chosen for the assessment is a major concern for both NICU physicians and other members of the NICU rehabilitation team [44, 45].

The data show that neither age nor gender significantly influences the ratings with any of the four scales (Table 2). They should not be fundamental factors in designing a rehabilitation program. There are, however, significant differences when the four scales tested are used with patients with different APACHE II scores.

The RASS, SAS and MAAS ratings were highly correlated with all types of subjects. The GCS ratings, however, correlated less well with the others (0.72–0.76, see Table 3). One explanation might be that the GCS was initially designed for coma rather than consciousness more generally as the other three were. With that in mind, the weaker correlation is reasonable if not acceptable. These results also agree well with those of a previous report by Nassar and his colleagues [46]. They found a relatively weak correlation between GCS ratings and those using other scales.

The stratified analysis of this study has shown that the four scales have similar inter-rater reliability. Previous studies have also found that all 4 scales have excellent agreement [37, 46]. All could easily be administrated in an NICU even by inexperienced professionals if they are properly trained. A study led by Brandl also found high reliability when a similar scale was used by ICU nurses without experience in assessment [47].

The three raters’ ratings also agreed well (see Table 4). The high correlation between any two sets of ratings further indicates the scales’ clinical applicability whether the rater is a senior physician or a trainee. It seems that clinical experience has not much impact on the scoring system as long as brief training has been provided. Clinical experience did, however, have some relationship with the judgments of consciousness made in this study. The correlation was low between the senior physician’s judgements and those of the trainee, especially in terms of the MAAS scores.

Although different scales have come to be applied globally in the last decade, few other than the GCS are ever applied to NICU patients in China. Related studies conducted in China have not given special attention to NICU rehabilitation. Today, most NICU staff in China remain unfamiliar with the scales tested here except for the GCS. Early intervention should then be based on reliable assessments of consciousness to formulate appropriate treatment plans [48]. This has probably been the first published study to quantify the validity and reliability the four scales with Chinese stroke subjects in an NICU. The results clearly indicate that all of the scales tested can be used easily and safely in the routine work with stroke patients in an NICU.

On the whole, the scales evaluated here are already being applied clinically in different countries around world [48, 49]. This study’s findings further testify to their reliability and feasibility when used with Chinese stroke survivors. However, considering all the subjects in this study were Chinese, whether the research protocol could be applied directly to another population elsewhere needs to be further verified. Even so, this study’s findings might be a useful reference for similar clinical situations in other countries and cultures.

## Conclusions

This study has confirmed that the RASS, SAS, MASS and GCS instruments all produce high univariate correlations among the ratings of very different raters, at least with Chinese stroke patients in an NICU.

## Data Availability

The datasets generated and analyzed during the current study are available upon the request from the corresponding author at yantb@mail.sysu.edu.cn.

## Abbreviations

APACHE II: Acute Physiology and Chronic Health Evaluation
CIs: Confidence Intervals
GCS: Glasgow Coma Scale
κ_w_: Weighted kappa
MAAS: Motor Activity Assessment Scale
NICU: Neurological Intensive Care Unit
RASS: Richmond Agitation-Sedation Scale
SAS: Sedation-Agitation Scale
